# Management of pregnant patients with pulmonary arterial hypertension

**DOI:** 10.3389/fcvm.2022.1029057

**Published:** 2022-11-10

**Authors:** Xiao Zhang, Zhao Huangfu

**Affiliations:** ^1^Department of Gynecology and Obstetrics, Beijing Hospital, National Center of Gerontology, Beijing, China; ^2^Institute of Geriatric Medicine, Chinese Academy of Medical Sciences, Beijing, China; ^3^Peking Union Medical College, Graduate School of Peking Union Medical College, Chinese Academy of Medical Sciences, Beijing, China; ^4^Department of Urology, Changhai Hospital, Naval Medical University, Shanghai, China

**Keywords:** pregnancy, pulmonary arterial hypertension, management, therapy, preconception management

## Abstract

Pregnant individuals with pulmonary arterial hypertension (PAH) have significantly high risks of maternal and perinatal mortality. Profound changes in plasma volume, cardiac output and systemic vascular resistance can all increase the strain being placed on the right ventricle, leading to heart failure and cardiovascular collapse. Given the complex network of opposing physiological changes, strict contraception and reduction of hemodynamic fluctuations during pregnancy are important methods of minimizing the risk of maternal mortality and improving the outcomes following pregnancy. In this review, we discuss the recent research progress into pre-conception management and the various therapeutic strategies for pregnant individuals with PAH.

## Introduction

Pulmonary arterial hypertension (PAH) is characterized by increased pulmonary artery pressure and vascular resistance, leading to increased right ventricular afterload, dysfunction or even failure which can be fatal (1). In 2016, it was estimated that a PAH prevalence of ~1% of the global population (2). Additionally, PAH prevalence in the United Kingdom has doubled in the last decade and is currently 125 cases/million person^1^. PAH among pregnant women confers high risks of mortality for both the mother and fetus. The adverse outcomes of PAH are exacerbated by the physiologic changes of pregnancy, which contribute to a high maternal mortality reaching 30–56% and a high neonatal mortality reaching 13% (3). With the improved treatment of PAH, maternal mortality has declined but remains high, ranging 11–25% (4–8). Given the high risk following pregnancy, appropriate contraception and reduction of hemodynamic fluctuations during pregnancy is key to reducing this risk of maternal mortality and improving pregnancy outcomes. In this review, we summarized the classification systems of PAH, strategies devised for preconception counseling and treatment strategies developed that are or will soon become available for pregnant women with PAH.

## Definition and classification

PAH is a heterogeneous disease that is characterized by an elevated mean pulmonary artery pressure of ≥20 mmHg at rest as per the 2022 European Society of Cardiology/European Respiratory Society (ESC/ERS) guidelines (1, 9). When echocardiographic tricuspid regurgitation velocity exceeds the threshold (>2.8 m/s), PAH should also be highly suspected according to the updated hemodynamic definition (10). It can be classified into the following five main groups based on the 2022 ESC/ERS guidelines: (i) Group 1, PAH; (ii) Group 2, PAH associated with left heart disease; (iii) Group 3, PAH associated with lung disease and/or hypoxia; (iv) Group 4, PAH associated with pulmonary artery obstructions; and (v) Group 5, PAH with unclear and/or multifactorial mechanisms (11) (Table 1).

**Table 1:** Updated classification from the 2022 ESC/ERS guidelines.

**1. PAH**
1.1 Idiopathic
1.1.1 Non-responders at vasoreactivity testing
1.1.2 Acute responders at vasoreactivity testing
1.2 Heritable
1.3 Associated with drug and toxins
1.4 Associated with:
1.4.1 Connective tissue disease
1.4.2 HIV infection
1.4.3 Portal hypertension
1.4.4 Congenital heart disease
1.4.5 Schistosomiasis
1.5 PAH with features of venous/capillary (PVOD/PCH) involvement
1.6 Persistent PH of the newborn syndrome
2. PH associated with left heart disease
2.1 Heart failure:
2.1.1 with preserved ejection fraction
2.1.2 with reduced or mildly reduced ejection fraction
2.2 Valvular heart disease
2.3 Congenital/acquired cardiovascular conditions leading to post-capillary PH
3. PH associated with lung diseases and/or hypoxia
3.1 Obstructive lung disease
3.2 Restrictive lung disease
3.3 Lung disease with mixed restrictive/obstructive pattern
3.4 Hypoventilation syndromes
3.5 Hypoxia without lung disease (e.g., high altitude)
3.6 Developmental lung disorders
4. PH associated with pulmonary artery obstructions
4.1 Chronic thrombo-embolic PH
4.2 Other pulmonary artery obstructions
5. PH with unclear and/or multifactorial mechanisms
5.1 Hematological disorders
5.2 Systemic disorders
5.3 Metabolic disorders
5.4 Chronic renal failure with or without haemodialysis
5.5 Pulmonary tumor thrombotic microangiopathy
5.6 Fibrosing mediastinitis

PAH, pulmonary arterial hypertension; PVOD, pulmonary veno-occlusive disease; PCH, pulmonary capillary hemangiomatosis; PH, pulmonary hypertension.

## Preconception management

### Contraceptives

The 2022 ESC/ERS guidelines recommended that all patients with PAH should be treated with contraception using a strict regimen (9). Although pregnancy may be feasible in some PAH patients with well-controlled disease, a low-risk profile, and normal or near-normal resting hemodynamics, for women with poorly controlled disease or women without fertility requirements, effective contraception is essential in view of the possible harm caused by contraceptive failure (12). The use of progesterone-only oral contraceptives, condoms, intrauterine contraceptive devices, or emergency post-coital contraception after intercourse is effective for PAH patients (9, 13). However, considering the obstacles of drug-drug interactions, potential contraindications to hormonal interventions (e.g., thromboembolism) and unreliability of contraceptive tools, a number of reports previously proposed that a combination of two or more contraceptive methods should be used, especially for patients with PAH treated with endothelin receptor antagonists (14–16). Tubal ligation is also an effective way to achieve permanent contraception, but patients have to face some risks of anesthesia accidents and complications since it is an invasive procedure. Therefore, tubal ligation can be performed at the same time when a woman is undergoing another surgery, such as cesarean section, reducing the risk of multiple surgeries. In addition, micro-insertion hysteroscopic sterilization is one of the effective contraceptive methods for patients with PAH due to its apparently lower surgical risk (17, 18).

### Preconception counseling

Because of high risk of adverse pregnancy outcomes and inherited heart defects (19, 20), the 2022 ESC/ERS guidelines recommended that women with PAH who are considering pregnancy or having already been pregnant should receive genetic counseling and shared decision-making at experienced medical centers (9). Fetal genetic testing should also be performed in pregnant women with known PAH-associated genetic mutations (21). Even though genetic testing is not performed, family members should be made aware of early signs and symptoms, to ensure that a timely and appropriate diagnosis is made (22). In fact, in addition to counseling on the risks associated with maternal mortality, patients at high risk should be informed about the option of avoiding pregnancy and therapeutic abortion. In such cases, alternatives such as adoption and surrogacy (if legal) may also be explored (9).

## Treatment

Although PAH patients have been required to take strict contraception, there are still some patients who do not take it because PAH has not been diagnosed before pregnancy, or still insist on pregnancy despite being aware of the higher pregnancy risks (23). Therefore, early drug intervention, comprehensive multidisciplinary assessment and co-management could become key factors in improving maternal and fetus outcomes (13, 16, 24). The principles of treatment for pregnant women with PAH are relieving clinical symptoms, enhancing exercise tolerance and improving pulmonary circulation hemodynamics. A summary of the Food and Drug Administration Pharmaceutical Categories in pregnancy and relevant PAH-associated therapeutic drugs are shown in Tables 2, 3, respectively.

**Table 2:** United States of America food and drug administration (FDA) pharmaceutical categories in pregnancy.

**Categories**	**Pregnancy risk**
A	Adequate and well-controlled studies have failed to demonstrate a risk to the fetus in the first trimester of pregnancy, and there is no evidence of risk in later trimester.
B	Animal reproduction studies have failed to demonstrate a risk to the fetus and there are no adequate and well-controlled studies in pregnancy women.
C	Animal reproduction studies have shown an adverse effect on the fetus and there are no adequate and well-controlled studies in humans, but potential benefits may warrant use of the drug in pregnant women despite potential risks.
D	There is positive evidence of human fetal risk based on adverse reaction data from investigational or marketing experience or studies in humans, but potential benefits may warrant use of the drug in pregnant women despite potential risks.
X	Studies in animals or humans have demonstrated fetal abnormalities and/or there is positive evidence of human fetal risk based on adverse reaction data from investigational or marketing experience, and the risk involved in use of the drug in pregnant women clearly outweigh potential benefits.

**Table 3:** Drugs and safety data.

**Classification**	**Drugs**	**FDA category**	**Placenta permeable**	**Transfer to breast milk (fetal dose)**	**Pre-clinical/clinical safety data**		**Method**	**Dose**	**Common adverse reactions**	**Reference**
					**Human data**	**Animal data**				
Anticoagulant	Aspirin	C	Yes	Yes (9–21%), potential toxicity, low dose (150 mg/d) may be indicated	Increase in gastroschisis in the first trimester and premature closure of the ductus arteriosus; cryptorchism; persistent pulmonary artery hypertension of the newborn in the third trimester; safe	Rats, mice, rabbits, monkeys and dogs: central nervous system and skeletal malformations, vascular defects	Oral	100 mg qd	Gastric ulcer, hemorrhage	(25–33)
	Fondaparinux	–	Yes (maximum of 10%)	Yes (rat)	Inadequate human data: use only when benefit outweigh risk	Rats/rabbits: subcutaneous doses up to 10 mg/kg/day in rats and at subcutaneous doses up to 10 mg/kg/day in rabbits revealed no evidence of impaired fertility or harm to the fetus	Inhalation	5–10 mg/d	Hemorrhage	(34–37)
	Heparin (unfractionated) (UFH)	B	No	No	Long-term use: less osteoporosis and thrombocytopenia than UFH, increased risk of maternal bleeding		Intravenous/inhalation	–	Hemorrhage, thrombocytopenia, anaphylaxis	(33, 38, 39)
	low molecular weight heparin (LMWH)	B	No	No	No increased risk of major developmental abnormalities	Rats/rabbits: no evidence of teratogenic effects of fetotoxicity	Inhalation	1 dose/d	Hemorrhage	(33, 40–44)
	Warfarin	D	Yes	Yes (maximum of 10%)	Coumarin embryopathy, bleeding		Oral	Adjust according to INR	Hemorrhage, gastrointestinal reaction, vasculitis	(33, 44–49)
Prostacyclin analog	Epoprostenol	B	Unknown	Unknown	Inadequate human data	Rats and rabbits: No impaired fertility or fetal harm	Intravenous	Starting dose 2 ng/kg/min; Target dose determined by tolerability and effectiveness; typical dose range at 1 year is 16–30 ng/kg/min, with wide individual variability	Headache, flushing, gastrointestinal reaction	(9, 24, 50–54)
	Iloprost	C	Unknown	Unknown	Inadequate human data: use only when benefit outweigh risk	Rats: shortened digits of the thoracic extremity in fetuses and pu-ps (these alterations are considered to be haemodynamic alterations in the fetoplacental unit and not teratogenic). Sprague- Dawley rats and monkeys: no such digital anomalies or other gross structural abnormalities. In Sprague-Dawley rats, iloprost clathrate (13% iloprost) significantly increased the number of non-viable fetuses at a maternally toxic oral dosage of 250 mg/kg/day, and in Han-Wistar rats it was found to be embryolethal in 15 of 44 litters at an i.v. dosage of 1 mg/kg/day	Inhalation	Starting dose 2.5 ug 6–9 times per day; Target dose 5.0 ug 6–9 times per day	Flushing, jaw pain, hypotension, cough, headache, flu-like syndrome, nausea, vomiting, syncope	(9, 24, 55–57)
	Treprostinil	B	Unknown	Unknown	Inadequate human data: use only if needed	Rabbits: increased incidence of fetal skeletal variations	Oral/ intravenous/ inhalation	(Starting dose 0.25 mg bid/0.125 mg tid; Target dose maximum tolerated dose up to 1,600 ug bid)/(Starting dose 1.25 ng/kg/min; (Target dose determined by tolerability and effectiveness; typical dose range at 1 year is 25–60 ng/kg/min, with wide individual variability)/(Starting dose 18 ug 4 times per day; Target dose 54–72 ug 4 times per day)	Pain at the infusion site, headache, flushing, gastrointestinal reaction, cough, flu-like syndrome, nausea, vomiting, tongue pain, syncope	(9, 24, 58–65)
Calcium channel blocker	Diltiazem	C	No	Yes	Possible teratogenic effects. Use only when benefit outweigh risk	Mice, rats, and rabbits: embryo and fetal lethality, and abnormalities of the skeleton, heart, retina, and tongue, reductions in early individual pup weights and pup survival, prolonged delivery, and increased incidence of stillbirths	Oral	Starting dose 60 mg bid; Target dose 120–360 mg bid	Edema, systemic hypotension, headache, nausea, dizzy, rash, fatigue	(9, 66–69)
	Nifedipine	C	Yes	Yes (maximum of 1.8%)	Tocolytic; sublingual application and potential synergism with magnesium sulfate may induce hypotension (mother) and fetal hypoxia. No teratogenic effects in the first trimester. Increased perinatal asphyxia, cesarean delivery, prematurity, and intrauterine growth retardation	Rodents, rabbits, and monkeys: embryotoxic, placentotoxic, teratogenic, and fetotoxic effects: stunted fetuses (rats, mice, and rabbits), digital anomalies (rats and rabbits), rib deformities (mice), cleft palate (mice), small placentas and underdeveloped chorionic villi (monkeys), embryonic and fetal deaths (rats, mice, and rabbits), prolonged pregnancy (rats; not evaluated in other species), and decreased neonatal survival (rats; not evaluated in other species)	Oral	Starting dose 10 mg bid; Target dose 20–60 mg bid/tid	Edema, systemic hypotension, dizzy, headache, nausea, fatigue, flushing	(9, 66, 69, 70)
Phosphodiesterase inhibitor	Sildenafil	B	Unknown	Unknown	Inadequate human data	Rats/rabbits: No teratogenicity, embryotoxicity, or fetotoxicity during organogenesis	Oral	20 mg tid	Headache, flushing, epistaxis, dyspepsia, rhinobyon, paropsia, backache, dizzy, rash	(24, 71–77)
Lipid-lowering drugs	Statins	X	Yes	Unknown	Congenital anomalies, but recent studies have found no significant increase in birth defects		Oral	–	Abdominal pain, constipation, flatulence, fatigue, headache	(66, 78, 79)

### General management

Pregnant women with PAH should avoid excessive activity but should still be encouraged to be active within symptom limits (16, 80). Whenever possible, lateral position should always be taken to minimize the compression of the inferior vena cava in the third trimester. Elastic support stockings can also be used whilst walking to avoid drastic changes in blood volume. In addition, appropriate supplementation of iron and folic acid should be encouraged to prevent anemia, whereas vaccination at least against influenza, Streptococcus pneumoniae and SARS-CoV-2 before pregnancy is recommended to enhance immunity (9, 81, 82).

For patients with PAH who decide to continue pregnancy, a standardized, individualized and realistic diagnosis and treatment plan should be formulated. They should receive regular and stringent evaluation and examination by clinicians from multiple disciplinary backgrounds during the pregnancy period (83). Prenatal check-ups should be performed every 2 weeks before 20 weeks and then once a week after 20 weeks of gestation. In addition to the routine obstetric examination, classification of the primary disease causing PAH, primary symptoms of PAH, cardiac function, echocardiography, 6-min walk test and biochemical indicators, including platelet count, hemoglobin, brain natriuretic peptide levels and arterial blood gas analysis, should also be focused upon (23). Since fetuses of pregnant women with PAH typically exhibit different degrees of growth restriction, ultrasound should be performed to examine the status of fetal growth at each obstetric examination (7, 13, 23). Hospitalization would be required if the condition worsens. Management by a multidisciplinary team is necessary for pregnant women with moderate to severe PAH (mWHO II-IV). This expert team should at least include a cardiologist, an ophthalmologist and an anesthesiologist with expertise in managing high-risk pregnant women with cardiac diseases. Furthermore, according to the unique scenarios posed by each patient, to produce a detailed delivery and emergency plan that includes the possibility of extracorporeal membrane oxygenation (ECMO) or transplantation, other specialists, including those who with extensive knowledge in pharmacy, cardiothoracic surgery, fetal medicine, neonatology, hematology, nursing and respiratory medicine, should be involved (1, 84). In fact, considering the high prevalence of symptoms of depression, anxiety and adjustment disorders in patients with PAH, empathic and hopeful communication is also crucial for physicians caring for pregnant women with PAH (9).

### Supportive therapy

#### Oxygen therapy

Oxygen inhalation is recommended when the peripheral oxygen saturation is < 90% or arterial oxygen partial pressure is < 60 mmHg. Maintaining the arterial partial pressure of oxygen at >70 mmHg and the oxygen saturation at >92% can reduce pulmonary vascular resistance, right-to-left shunt and the incidence of low-birth-weight infants (85). Nocturnal oxygen therapy should be considered in case of sleep-related desaturation (86). Oxygen inhalation should also be considered when pregnant patients with PAH travel by air, especially those with New York Heart Association (NYHA) class III-IV PAH or with the arterial partial pressure of oxygen < 8 kPa (87, 88). Although oxygen administration reduces pulmonary vascular resistance and improves exercise toleration in patients with PAH, there are no data to suggest that long-term oxygen therapy has sustained benefits on the course of the disease (9).

#### Diuretics, cardiotonics and antiarrhythmics

Diuretics can relieve the symptoms of decompensation following right heart failure. Torasemide or furosemide are options of loop diuretics during pregnancy. However, spironolactone should be avoided during the first trimester because due to its antiandrogenic effects (13, 66, 89, 90). During the use of diuretics, regular monitoring of kidney function and serum electrolytes is necessary to avoid intravascular volume depletion and further decline in cardiac output and systemic blood pressure.

Cardiac agents, including digoxin or milrinone, can improve cardiac output in patients with PAH, although their long-term efficacy remains unclear. In addition, maintenance of sinus rhythm is important, because atrial fibrillation and supraventricular tachycardia are associated with the occurrence of right heart failure and subsequent mortality (91). Intravenous adenosine is the priority treatment method for acute episodes of paroxysmal supraventricular tachycardia (PSVT). By contrast, β-adrenergic receptor blockers, with the exception of atenolol, are first-line drugs for PSVT prevention. Electrical cardioversion is also recommended when patients with atrial fibrillation become hemodynamically unstable or if they are considered to be at great risk of mortality (92). Ibutilide or flecainide injections may be considered to eliminate atrial fluttering and atrial fibrillation in PAH patients with structurally normal hearts (93). By the same token, since patients with congenital heart disease do not typically tolerate atrial flutter effectively, electrical cardioversion should be performed to restore sinus rhythm (94). Idiopathic right ventricular outflow tract tachycardia is the most common type of ventricular tachycardia, which requires β-adrenergic receptor blockers, verapamil or other antiarrhythmic prophylactics. If drug treatment fails, catheter ablation would then be needed. If possible, catheter ablation should be delayed until the second trimester and performed at a well-equipped medical center containing experts with extensive experience (95, 96). In addition, occasional sinus bradycardia may be associated with the supine hypotensive syndrome. Symptomatic bradycardia should be treated with changes in maternal position, following which temporary pacemaker should be installed if clinical symptoms persist (97, 98). Temporary pacemakers are usually not required in hemodynamically stable patients but are recommended during labor in women at risk of bradycardia or those with a history of syncope (97). Implantable cardioverter defibrillators should also be considered before pregnancy in patients at high risk of sudden cardiac death (80, 99).

#### Anticoagulant therapy

Pregnant women frequently present with hypercoagulability, which can increase the risk of thrombosis. Anticoagulants are recommended for reducing the risk of thrombosis if cardiopulmonary dysfunction was detected in the pregnant individual with PAH. The choice of anticoagulant drugs should be comprehensively considered based on the individual involved, gestational stage, risk of maternal bleeding and teratogenic effects (15, 100). Due to low molecular weight heparin (LMWH) having lower reported levels of impact on the fetus and lower risks in terms of osteoporosis, it is considered to be the priority drug for preventing and treating venous thromboembolism during pregnancy and puerperium. However, the preventive effect of valve thrombosis conferred by LMWH is weak (38). If LMWH or unfractionated heparin (UFH) is used during pregnancy, then it is recommended to calculate the initial dose according to the maternal weight in the first trimester (8–10 gestational weeks). The dose should be subsequently adjusted by monitoring the anti-factor Xa levels or partial thromboplastin time weekly until the 4- to 6-h peak anti-Xa levels reached 0.6–1.2 IU/ml (1, 101). Fondaparinux may even be considered for PAH patients if they are allergic to LMWH. Warfarin can pass through the placenta and may exert dose-dependent teratogenic effects, which can lead to “Fetal Warfarin Syndrome” (102). Therefore, warfarin should be used at limited dosages or be replaced with LMWH during the first trimester. The dosage of warfarin needs to be limited to < 5 mg/d and the international normalized ratio (INR) should be adjusted to 1.5–2.0 during the second and third trimesters. However, the safety profile of warfarin in pregnant women remains controversial and should therefore be avoided where possible during pregnancy (40, 45, 103–106). In addition, high-quality evidence supporting the safety and efficacy of novel oral anticoagulants, such as dabigatran, rivaroxaban and apixaban, in pregnant women with remain elusive obstructing their recommendation for use (107, 108). A meta-analysis of 339 pregnant women on new direct oral anticoagulants previously revealed that 22.2% suffered from miscarriages, whilst 3.6% had fetuses with skeletal and facial deformities (109). In particular, all patients had to terminate oral anticoagulation during the first 2 months of pregnancy, suggesting that the novel oral anticoagulation regimens used during pregnancy may be associated with higher risks of miscarriage and birth defects (110).

Oral anticoagulants should be replaced with LMWH or UFH 3–5 days before delivery, which would render it safer for cesarean sections. However, LMWH should be stopped 12–24 h before delivery, whereas UFH should be stopped >4–7 days before delivery. Protamine antagonism can be carefully applied for pregnant women requiring the emergency termination of pregnancy but did not stop heparin treatment. By contrast, warfarin can be antagonized by vitamin K1 antagonism (15, 111–113). If there is no obvious bleeding for 24 h after delivery, anticoagulation therapy can be resumed. It should however be noted that patients who were treated with warfarin prior should instead be administered with LMWH and have the INR monitored for several days after delivery. LMWH can then be stopped after warfarin comes into effect.

### PAH-specific therapy

Currently available drugs targeting the pathological pathways underlying PAH did not result in the complete reversal of the condition but have been found to reduce pulmonary artery pressure and alleviate clinical symptoms. For patients with stable hemodynamics, routine use of vasodilators and other drugs that can interfere with hemodynamics is not recommended. However, for patients with unstable hemodynamics, addition of pulmonary vasodilators on cumulatively on the existing general treatment regimens can significantly reduce the risk of maternal mortality and adverse pregnancy outcomes (14, 114). These vasodilators include calcium channel blockers (CCB) (4, 6, 115), prostaglandins and associated analogs (7, 50, 51, 55, 116) and phosphodiesterase inhibitors (PDE-I) (117), but no endothelin receptor antagonists due to the teratogenic potential (118, 119). Pieper et al. suggested that pharmacological treatment of PAH should be started at ≥3 months before delivery, because the optimal effects did not appear until 3 months later (14). However, since the use of pulmonary vasodilators in pregnant women may endanger the safety of the fetus, their application must be evaluated with their standard formulations and usage requiring additional support by high-quality evidence-based research.

#### Prostaglandins and analogs

Prostacyclin can inhibit smooth muscle proliferation, reverse vascular remodeling, expand pulmonary and systemic circulation, enhance right ventricular function and exert anti-platelet effects (120). However, thrombocytopenia and bleeding-related complications may occur after the patients are treated with anticoagulants and prostacyclin at the same time. Therefore, blood routine and coagulation function parameters should be monitored closely (45). Indications of initiation use of prostaglandins are typically patients with WHO FC class III-IV PAH or impaired right ventricular function (88, 121–123).

At present, epoprostenol is the most widely used targeted drug in pregnant women with PAH (62%), which is also the first-line drug for the treatment of pregnancy with PAH or Eisenmenger syndrome (52, 124–126). Epoprostenol can improve exercise capacity, quality of life and survival rate (120). Due to its slow onset time and short half-life, it is recommended to commence treatment with this drug ≥8 weeks prior to delivery through the central venous catheter (120). It can also be used postpartum for preventing pulmonary hypertension crisis and right heart failure (127, 128). There is a lack of data and experience on the safety of this drug on the fetus, but several studies showed that it did not cause fetal malformation or intrauterine growth restriction in the third trimester (55, 116). Adverse reactions associated with the long-term use of epoprostenol may include facial flushing, headache, diarrhea, abdominal pain, uterine contractions, and postpartum hemorrhage. However, dosage reduction is only required when the adverse reactions become severe.

Iloprost is an inhaled prostacyclin analog that is frequently used to relieve dyspnea during surgery. The onset time is generally 15–20 min and the duration of drug effects is 1–2 h (125, 127, 129). Compared with intravenous administration, short-term application of aerosol iloprost inhalation tend to be more effective in reducing pulmonary arterial pressure (6, 7, 55). At present, there is no evidence indicating that inhaled iloprost can cause maternal mortality or congenital fetal anomalies. However, it was indicated that inhalation of iloprost within 24 weeks of gestation and perinatal period can significantly reduce the risk of fetal malformations and mortality (55, 114, 130). It is noteworthy that intravenous epoprostenol should be applied if the disease worsens after inhaling iloprost (52, 114). Inhaled prostaglandins are generally used for patients with less severe symptoms of PAH due to the short half-time caused by intermittent nebulized administration, which may lead to rebounds in pulmonary artery pressure (13, 55). However, it is a sign of serious condition if patients with PAH take iloprost before conception.

Treprostinil can be administered through various means, including subcutaneous infusion, intravenous and oral administration (131). This drug can be used for patients with WHO cardiac function class II-IV or NYHA III-IV PAH. It has been reported to significantly improve the 6-min walking distance, peripheral blood oxygen saturation and cardiac function (132). However, some patients (about 8%) refused to use this drug due to the infusion-site pain (133).

#### CCBs

A number of pregnant women with PAH may benefit from CCBs. However, CCBs are only typically used in patients whose acute pulmonary vasodilation test results are positive (4, 6, 85, 115). Diltiazem is the first choice CCBs for patients with fast heart rhythm, whereas nifedipine is used for patients with slow heart rhythm. The minimum dose should be used at first with the dose increasing gradually according to blood pressure, cardiac rhythm, heart rate, electrocardiogram results and clinical symptoms until the maximum tolerated dose is reached. If the right heart structure and function remain at physiological levels and the pulmonary arterial pressure is normal or close to normal (mean pulmonary arterial pressure ≤30 mmHg and pulmonary vascular resistance < 4 WU) after receiving CCB therapy, it can be adjudged that the patient is continuously sensitive to CCBs, meaning that it can be applied chronically. If there is no satisfactory response, additional PAH therapy should be instituted. However, various studies showed that CCBs are not able to mediate long-term vasodilation effects on patients with PAH, where exposure to CCBs in the third trimester may increase the risk of neonatal seizures (Odd ratio = 3.6) (134, 135). Therefore, it is necessary to consider the gradual conversion from CCBs to prostaglandins or analogs or PDE-I if cardiac function and pulmonary artery pressure are not maintained at normal levels (127).

#### PDE-I

PDE-5 inhibitors are the priority drug for women with WHO cardiac function class I-II PAH or normal right ventricular function (5, 71, 136). The expression levels of PDE-5 in the right ventricular myocardium are frequently found to be increased in patients with PAH. PDE-5 inhibitors can reverse this change to relax vascular smooth muscles and reduce the pulmonary artery pressure (6). In addition, the use of PDE-5 inhibitors does not change lactate levels or pH levels in the fetus. Sildenafil is the most extensively applied PDE-5 inhibitor. It has been previously found that sildenafil can significantly improve the clinical symptoms, cardiac function, hemodynamic indices and pregnancy outcomes (111). It can also increase the rate of successful vaginal delivery and reduce the incidence of premature births or infants with low birth weights (130, 136, 137). If cardiac function continues to deteriorate and the pulmonary vascular resistance increases after oral sildenafil treatment, inhaled iloprost may be considered to relieve symptoms (136, 137). In addition, a previous study indicated that five pregnant women with PAH who received PDE-5 inhibitors and prostacyclin intravenously did not show significant maternal and infant complications during long-term follow-up at the breastfeeding stage (84).

Although sildenafil is widely used, its safety during requires further study. In 2021, a study focusing on 77 infants born to women who were treated with PDE-5 inhibitors during pregnancy found that nine infants were premature, six had small statures for their gestational age, five scored < 8 in their 5-min appearance, pulse, grimace, activity and respiration tests, 18 were admitted to the neonatal intensive care unit and eight were diagnosed with respiratory and cardiovascular diseases (138). Furthermore, there is no clinical evidence to support the efficacy of vardenafil and tadalafil in pregnant women with PAH at present.

#### Statins

In addition to hypolipidemic effects, statins also have reported anti-inflammatory, anti-proliferative and apoptotic effects. A large number of clinical and experimental studies revealed that statins can reduce the pulmonary artery pressure and improve pulmonary vascular remodeling by promoting apoptosis, inhibiting cell proliferation, anti-inflammatory effects, inhibiting Rho kinase signaling and endothelin-1 release (139–142). Although statins also did not increase the rate of birth defects or the rate of fetal teratogenicity, the sample size was relatively small (78). Although statins are generally not recommended during pregnancy and lactation at present, they may be used for the long-term treatment of postpartum PAH after lactation (66, 79).

### ECMO

ECMO is an advanced respiratory and circulatory support technology that has been proposed for patients with acute reversible respiratory failure (143). ECMO can maintain the oxygenation status of patients with PAH when cardiac function is decompensated (144, 145). Various case reports suggested that ECMO treatment in the majority of pregnant women with PAH conferred favorable pregnancy outcomes (146–148). However, it has also been reported that patients with PAH receiving ECMO did not survive beyond 3 months after delivery due to severe right heart failure (149). In addition, the continuous operation of ECMO requires systemic anticoagulation, where the continuous oozing of the surgical incision may lead to severe coagulation dysfunction (150, 151). Due to the small sample size reported thus far, further studies on larger sample sizes are warranted.

### Operative therapy

PAH secondary to a known cardiopulmonary disease requires the aggressive treatment of primary disease, with procedures including biventricular pacing and cardiac operation (85). Cardiac surgery under cardiopulmonary bypass may be considered for patients who have responded to drug therapy but have opportunities for surgery. Surgery should be performed between 13 and 28 weeks of gestation. If the gestational week is >26 weeks, then cesarean section can be considered to terminate the pregnancy before performing the cardiopulmonary bypass operation. If the gestational week is >28 weeks, cardiac surgery can be considered after vaginal delivery. However, the risk of maternal and fetal mortality remains high during cardiopulmonary bypass operation. Cardiac surgery is recommended only when drug or other forms of interventional therapy fails and the pregnant individual is in mortal danger (1, 88, 152).

For patients with chronic thromboembolic PAH, pulmonary endarterectomy is a possible curative method, although not all patients are suitable for this form of surgical treatment. Chronic thromboembolic PAH may also require postpartum thrombectomy or lung transplantation (146). High-risk patients who decide to continue pregnancy should be promptly evaluated for lung transplantation (6).

## Conclusions

PAH is considered to be an irreversible disease. Strict contraception or early termination of pregnancy is recommended for PAH of any severity. However, effective early management is important for improving the prognosis for pregnant women with PAH first discovered during pregnancy or strongly required to continue pregnancy. Strategies, such as anticoagulation therapy, PAH-specific therapy and operative therapy, should be considered. Since pregnancy with PAH is a rare condition, the majority of relevant studies are retrospective studies based on case reports and case series analysis, which have low levels of evidence-based medical evidence. Therefore, additional prospective multi-center studies should be conducted in the future to optimize the clinical management strategies for pregnant women with PAH.

## Author contributions

XZ and ZH conducted the study conceptualization, writing, and review of the manuscript. Both authors contributed to the review and approved the submitted version.

## Conflict of interest

The authors declare that the research was conducted in the absence of any commercial or financial relationships that could be construed as a potential conflict of interest.

## Publisher's note

All claims expressed in this article are solely those of the authors and do not necessarily represent those of their affiliated organizations, or those of the publisher, the editors and the reviewers. Any product that may be evaluated in this article, or claim that may be made by its manufacturer, is not guaranteed or endorsed by the publisher.

## Footnotes

^1^NHS Digital. *National Audit of Pulmonary Hypertension 10th Annual Report, Great Britain, 2018-19*. Available online at: https://digital.nhs.uk/data-and-information/publications/statistical/national-pulmonary-hypertension-audit/2019 (accessed October 19, 2022).
